# The Inevitability of Ethnocentrism Revisited: Ethnocentrism Diminishes As Mobility Increases

**DOI:** 10.1038/srep17963

**Published:** 2015-12-08

**Authors:** Soham De, Michele J. Gelfand, Dana Nau, Patrick Roos

**Affiliations:** 1 Department of Computer Science, University of Maryland, College Park, MD, USA; 2 Department of Psychology, University of Maryland, College Park, MD, USA; 3 Department of Computer Science and Institute for Systems Research, University of Maryland, College Park, MD, USA

## Abstract

Nearly all major conflicts across the globe, both current and historical, are characterized by individuals defining themselves and others by group membership. This existence of group-biased behavior (in-group favoring and out-group hostile) has been well established empirically, and has been shown to be an inevitable outcome in many evolutionary studies. Thus it is puzzling that statistics show violence and out-group conflict declining dramatically over the past few centuries of human civilization. Using evolutionary game-theoretic models, we solve this puzzle by showing for the first time that out-group hostility is dramatically reduced by *mobility*. Technological and societal advances over the past centuries have greatly increased the degree to which humans change physical locations, and our results show that in highly mobile societies, one’s choice of action is more likely to depend on what individual one is interacting with, rather than the group to which the individual belongs. Our empirical analysis of archival data verifies that contexts with high residential mobility indeed have less out-group hostility than those with low mobility. This work suggests that, in fact, group-biased behavior that discriminates against out-groups is not inevitable after all.

Nearly all major conflicts across the globe, both current and historical, are characterized by individuals defining themselves and others in terms of their group membership. Substantial empirical evidence supports people’s tendency to favor in-group members and show hostility towards out-group individuals^1^,^2^,^3^,^4^,^5^. From an evolutionary perspective, numerous studies have shown how in populations comprised of various groups, group-biased behavior that discriminates or is hostile against out-groups evolves or emerges readily and dominantly^6^,^7^,^8^,^9^,^10^,^11^,^12^. Since humans are social beings who establish and define groups constantly, the development of out-group hostility and resulting group conflict might thus seem inevitable.

In a puzzling contrast, statistics have shown that violence and out-group conflict have actually declined dramatically over the past few centuries of human civilization, suggesting out-group hostility is not inevitable after all^13^,^14^. What factors might lead to such a decrease in conflict? Evolutionary game-theoretic models can shed light on this question by exploring how various factors affect the emergence and maintenance of individuals’ behaviors relating to group conflict.

Our evolutionary game model builds on a prior model developed in Hammond and Axelrod’s pioneering work^6^ on the evolution of ethnocentrism, and used, for example, in Hartshorn, *et al.*^12^. In their model, agents had perceivable group tags, played one-shot Prisoner’s Dilemma games with their neighbors, and could behave differently toward in-group members than out-group members. Each agent’s inherited traits included a group tag, an action (cooperate or defect) to use with in-group members, and a similar action to use with out-group members. Thus there were four possible strategies: *Cooperate* with both in-group and out-group members; *Defect* against both in-group and out-group members; *Ethnocentric* (cooperate with in-group members, defect against out-group members); *Traitorous* (defect against in-group members, cooperate with out-group members).

Using their model with four different groups (or group tags), we have replicated their result showing that after a period in which Cooperative agents are briefly abundant, evolutionary pressure leads to a predominance of Ethnocentric agents. Defectors and Traitors never establish themselves (see the Supplementary Material for details).

Since the agents in that model conditioned their actions only on the group tags, they were in effect *group-entitative*. That leaves open the question whether there are conditions under which *individual-entitative* agents—agents that base their actions on knowledge of individuals *per se* rather than group tags—may be able to exist and perhaps even be favored by evolutionary pressures.

Moreover, that model does not incorporate *mobility*. Research in cultural psychology has demonstrated large empirical differences in residential mobility around the globe with important psychological consequences^15^,^16^. Researchers have shown that in high-mobility contexts, individuals change relationships often; they form new relationships and sever unwanted relationships with great ease^17^,^18^. In such contexts, having a broad network of weak ties and being open toward strangers (with whom it might be valuable to form relationships) is highly adaptive. Indeed, Oishi, *et al.*^18^ observe that in highly mobile contexts, “since it is hard to keep track of behaviors of many strangers whom one meets, one needs to carefully avoid being associated with defectors or free-riders in order to exploit the greatest possible relational benefit” (p. 228). Thus, individuals are more likely to adopt strategies that try to evaluate the “trustworthiness and worth”^18^ of others in highly mobile contexts, i.e., adopt *individual-entitative* strategies. On the other hand, in low-mobility contexts, individuals have far fewer opportunities to form new relationships, and severing existing relationships can have extreme adverse effects such as being ostracized from one’s only social circle^18^, causing “the existential, social, and psychological death of the individual” (p. 755)^19^. Based on these theories we would predict that group-entitative behavior and associative ethnocentrism is adaptive in low mobility societies, yet it is maladaptive in high-mobility contexts, where individual-entitative strategies would be evolutionarily favored.

We have run extensive new evolutionary simulations, augmenting the prior model to include individual-entitative strategies and mobility; and our results show that the evolution of ethnocentrism is driven by low mobility. Indeed, our subsequent empirical analysis of archival data verifies that contexts with high residential mobility have less out-group hostility than those with low mobility.

In our evolutionary game model, agents are arranged on a toroidal (wrap-around) grid, so that every node on the grid is connected to 4 neighboring nodes). Initially the grid is empty. The sequence of events at each time step is shown in Fig. 1; these are the same as in Hammond and Axelrod’s paper^6^ except for the *Mobility* stage, which is new. For additional details, see the Methods section.

The agents’ strategies are similar to those in Hammond and Axelrod’s model^6^, where agents can distinguish between in-group and out-group members by observing the group tags. Hence agents’ strategies can be conditioned on whether they are interacting with in-group or out-group members. In addition, in our model, each agent’s strategies can be conditioned on the past history of other agents. Each agent can either be group-entitative or individual-entitative, and this is an inherited trait.

A *group-entitative* agent *i* ignores individual identities. Its actions toward an agent *j* depend only on its last encounter with *anyone in j’s group*. It has two possibly different strategies: one for in-groups and another for out-groups. Each of those strategies is one of the following: *AllC* (always cooperate), *AllD* (always defect), *TFT* (Tit-for-Tat: play whatever action the opponent played in *i*’s last interaction with anyone from *j*’s group), or *OTFT* (play the opposite of what *TFT* would play). For details about *i*’s behavior during its first encounter with each group, see the Supplementary Material.

An *individual-entitative* agent *i* ignores other agents’ group tags; *i*’s action toward *j* depends only on its last encounter *specifically with j*. Thus *i* has one of the above four strategies, except that *TFT* and *OTFT* depend on *i*’s last interaction with *j* specifically, rather than someone in *j*’s group.

To model *mobility*, there is a probability *m* with which, at the beginning of each iteration, an agent moves to a randomly chosen empty spot in the network. Thus a high value of *m* represents a highly mobile population, while a low value of *m* represents a population with low mobility. We vary *m* from 0 to 0.08 in our experiments. It is important to note that a mobility probability of 0.08 is quite high: it means that on average, 8% of the population move to different locations on each iteration—a substantial amount of movement even for small values of *m.* At higher levels of mobility (*m* > 0.1), cooperation breaks down in a society, and the majority of the population starts defecting—and thus is not representative of any stable society around the world (see the Supplementary Material for details).

## Results

**Figure 2:**
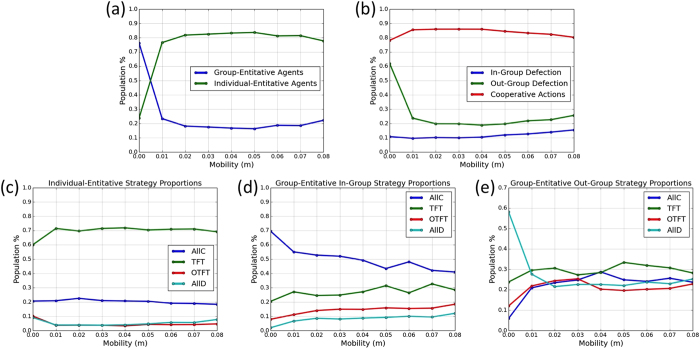
Proportions of actions and strategies as a function of mobility, after 30,000 iterations. Each data point is an average of 100 simulation runs. The plots show the proportions of (**a**) the group-entitative and individual-entitative agents, (**b**) the actions played by the agents, (**c**) the strategies of the individual-entitative agents, and (**d**) the in-group and (**e**) out-group strategies of the group-entitative agents.

Figure 2 shows our results after letting the populations evolve for 30,000 iterations. Without mobility (i.e., *m* = 0), group-entitative agents comprise 75% of the population. These agents’ strategies are predominantly out-group hostile (*AllD*) and in-group cooperative (*AllC*). This is reasonably consistent with Hammond and Axelrod’s model^6^, but notice that even when *m* = 0, individual-entitative agents comprise about 25% of the population.

As mobility increases, the evolutionary pressures shift to favor individual-entitative agents. For *m* > 0.02 they comprise about 80% of the population, and about 70% of them play *TFT*. Thus, the evolutionary dominance of group-entitative and ethnocentric strategies is thwarted by mobility.

**Figure 3:**
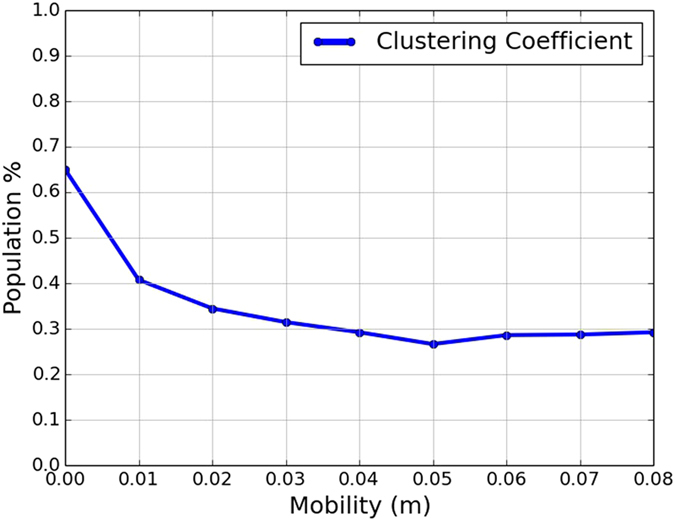
Clustering coefficients after 30,000 iterations, for varying mobility values. Each data point is an average of 100 individual simulation runs. The degree of clustering decreases with mobility. For details on the metric used, see the Methods section.

The reason why low mobility favors group-entitative strategies while higher mobility favors individual-entitative strategies is related to the clustering of group members (Fig. 3). With low mobility, groups tend to cluster together heavily; hence agents interact primarily with in-group members. Thus the ethnocentric strategy (i.e., group-entitativity with in-group cooperation and out-group-hostility) is effective and profitable in terms of payoff. Under higher mobility, however, agents are less clustered by group membership, hence more likely to interact with out-group members, hence cannot rely on high payoffs from in-group interactions. Furthermore, group-entitative strategies are less effective because different individuals within a group are much less likely to have the same strategy. This favors the individual-entitative Tit-for-Tat (*TFT*) strategy (see the Methods section for more information on the clustering coefficient).

**Figure 4:**
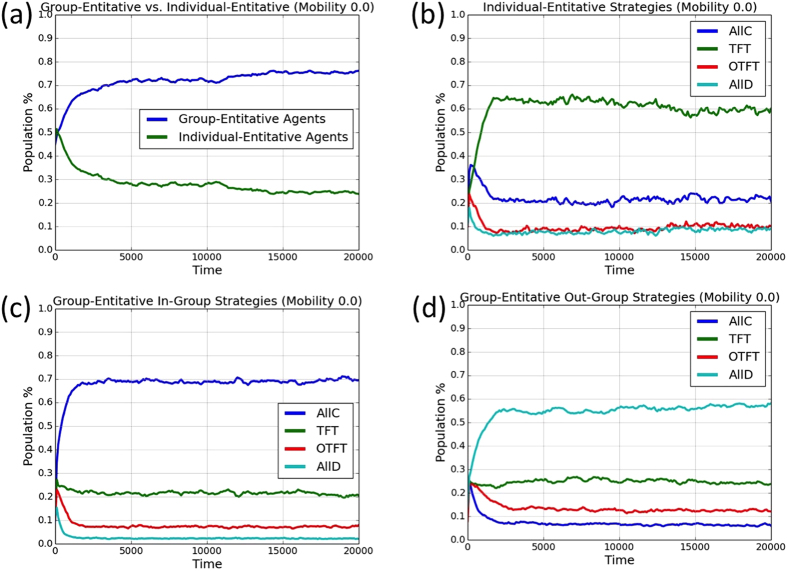
Single simulation run for 20000 generations with no mobility (*m* = 0). (**a**) Proportions of group-entitative and individual-entitative agents. (**b**) Relative proportions of the individual-entitative agents’ strategies; Relative proportions of the group-entitative agents’ (**c**) in-group and (**d**) out-group strategies.

**Figure 5:**
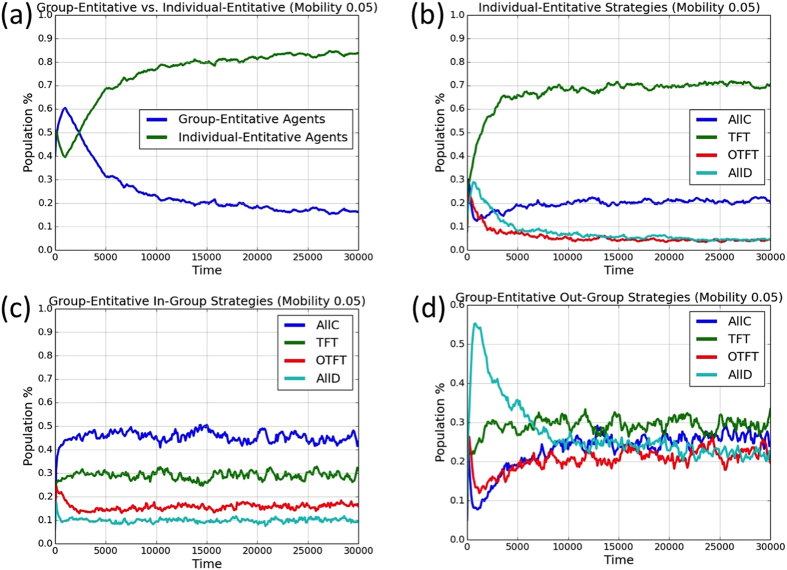
Single simulation run for 30000 generations with mobility probability *m* = 0.05. (**a**) Proportions of group-entitative and individual-entitative agents. (**b**) Relative proportions of the individual-entitative agents’ strategies; Relative proportions of the group-entitative agents’ (**c**) in-group and (**d**) out-group strategies.

To illustrate the evolutionary trajectories that led to the results reported in the main paper, Figs 4 and 5 show representative evolutionary trajectories for single simulation runs. In Fig. 4, there is no mobility. Group-entitative agents quickly become a majority, and most of them are ethnocentric (in-group cooperative and out-group hostile). In Fig. 5, the mobility probability is *m* = 0.05. Individual-entitative agents evolve to become a majority, with most of those agents playing Tit-for-Tat (*TFT*).

To illustrate robustness of the results with our model, we also performed a series of experiments where we initialized the population on the grid to have high clustering of group-entitative or individual-entitative agents (instead of starting out with an empty grid). In each case, we notice that the results are the same as when we start out with an empty grid, i.e., group-entitative agents dominate under no mobility and individual-entitative agents dominate under higher values of mobility. This is due to the exploration dynamics (or mutation phase) in our model. The exploration dynamic has been shown to be a key aspect of evolutionary game-theoretic models for cultural evolution^20^,^21^, and this ensures that our model remains robust to the initial conditions of the grid. Please see the Supplementary Material for more details.

### Empirical Analysis

In order to complement these modeling efforts, we also gathered data to test the notion that mobility relates to lower ethnocentrism. We analyzed data from the U.S. Census Bureau^22^,^23^ that provides measures of mobility in the U.S. 50 states (defined as the percentage of people born in the state of residence; reverse scored, with higher scores being reflective of higher mobility) and data from the DDB Needham Life Style Survey^23^. We found that mobility was positively correlated with responses to the question “I am interested in the cultures of other countries” (*r* = 0.614, *p* <0 .001), and negatively correlated with responses to questions regarding ethnocentrism (e.g., Americans should always buy American products, *r* = –0.654, *p* < 0.001; The government should restrict imported projects, *r* = –0.578, *p* < 0.001).

In addition, states that have higher mobility also have higher openness, one of the big five personality dimensions, which is associated with breadth of experience and interest and interest in new ideas and other cultures (*r* = 0.321, *p* = 0.023)^24^.

## Discussion

The evolution of cooperation has been of great scientific interest in many disciplines; and to date, many evolutionary and empirical studies have found that in-group-favoring and out-group hostile behaviors are common. This has caused much concern that group conflict and ethnocentrism is an inevitable threat on our planet. We integrate research on group conflict with human mobility^25^,^26^,^27^,^28^,^29^,^30^,^31^, and show for the first time that the evolution of ethnocentrism and group entitative behavior is thwarted by high mobility. As mobility is rapidly changing around the globe^18^, this work predicts that group conflict will continue to decrease, in line with Pinker’s historical analysis^13^,^14^.

Mobility is an important and well-studied topic in cultural psychology^26^,^27^,^28^,^29^,^30^. Low mobility leads to conditions where interacting individuals are likely to be reproductively related and this has been shown to be important in the evolution of cooperation^25^,^31^. In our model, we find mobility plays a crucial role in the evolution of ethnocentrism in a society. More specifically, we establish that low mobility leads to in-group cooperation and out-group hostility. High mobility, on the other hand, leads to more individual-entitative behavior, where agents take actions based on the specific individuals with whom they interact, rather than the groups to which those individuals belong.

Another unique aspect of our model is that we allow for agents to have memory of previous actions played by other agents and the possibility of individual-entitative agents, where agents take actions based on the individual they are playing against rather than their tag. In a society with high mobility, agents would be moving to different parts of the grid, which leads to low clustering of agents belonging to the same group. Thus, agents with group-entitative strategies suffer, which leads to the evolution of individual-entitative strategies, with strategies like Tit-for-Tat gaining prominence. Under low mobility, on the other hand, agents of the same group cluster together much more, and simple group-entitative strategies like in-group cooperative and out-group hostility gain prominence.

It would be fruitful to incorporate mobility into other evolutionary game-theoretic models of conflict in future research. Moreover, since mobility in a society could be motivated by multiple factors that could have divergent effects, future models of mobility and ethnocentrism should incorporate these motivations. Mobility might reduce ethnocentrism when agents move for economic reasons, but mobility might not reduce ethnocentrism if agents move primarily to be among other in-group members. In all, our work shows for the first time that mobility is a critical factor that affects ethnocentrism with important implications for theory and policy.

## Methods

### Evolutionary dynamics of our model

**Figure 6:**
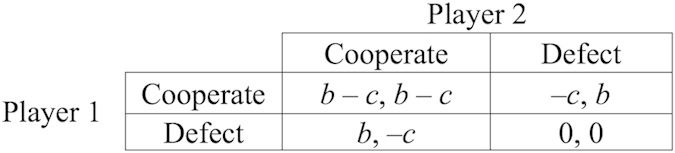
Prisoner’s Dilemma payoff matrix used in both our model and the previous one.

Here is a more detailed version of the sequence of steps in our evolutionary game-theoretic model:

1. *Birth*: One agent with a random strategy appears at a random empty site, if such a site exists.
2. *Base Payoff*: Each existing agent receives the base payoff from the environment; we use a base payoff of 0.12 throughout our experiments.
3. *Interaction Payoff*: Each agent plays a game with each of its neighboring agents on the grid, receiving payoffs according to the game definition. The game played by the agents is the canonical 2-player cooperation/defection dilemma (Fig. 6), with the benefit of cooperation *b* = 0.03 and the cost of cooperation *c* = 0.01. In this phase, the action chosen by an agent in each game depends on the type of agents playing that game (i.e., group-entitative or individual-entitative agent) as well as the type of strategy being used by that agent.
4. *Fitness*: Each agent is assigned fitness equal to the agent’s accumulated payoff.
5. *Reproduction*: In random order, each agent is given a chance to reproduce with probability equal to its fitness. If an agent gets a chance to reproduce, it places an offspring in a randomly chosen empty site in its neighborhood, if such a site exists. The offspring has the same traits as its parent, with a mutation rate of *μ* = 0.005 per trait.
6. *Death*: Each agent has a probability *d = 0.1* of dying. If an agent dies, it is removed from the grid.
7. *Mobility:* Each agent has a probability of *m* of moving to a randomly chosen empty spot on the grid.

### Clustering coefficient

A clustering coefficient is a metric for measuring the amount of clustering of nodes in a graph. We can measure the clustering of group tags by comparing the group tags of the agents at neighboring locations. For each location (*x, y*) on the torus grid we consider four triples, each consisting of (*x, y*) and a pair of adjacent neighboring locations:

1. location (x, y), the neighbor above it, and the neighbor to its left;
2. location (x, y), the neighbor above it, and the neighbor to its right;
3. location (x, y), the neighbor below it, and the neighbor to its left;
4. location (x, y), the neighbor below it, and the neighbor to its right.

Our clustering coefficient is the total number of triples that contain three agents with the same group tag, divided by the total number of triples in the grid. For a torus grid of size *N* × *M*, the denominator in our metric, i.e., the number of total triplets, is simply 4*NM*. The clustering coefficient lies in the range from 0 to 1, and is higher when agents of the same tag cluster together on the grid, while being small when there is less clustering of agents of the same tag.

## Additional Information

**How to cite this article**: De, S. *et al.* The Inevitability of Ethnocentrism Revisited: Ethnocentrism Diminishes As Mobility Increases. *Sci. Rep.* **5**, 17963; doi: 10.1038/srep17963 (2015).

## Supplementary Material

Supplementary Information

## Figures and Tables

**Figure 1:**
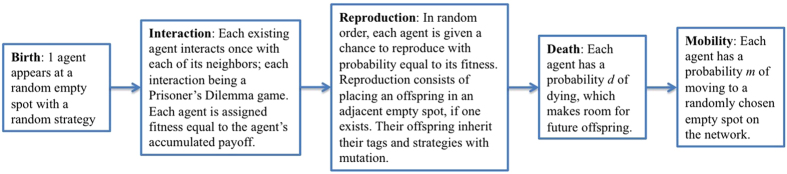
Sequence of events at each time step in our evolutionary game-theoretic model. The sequence of steps are the same as in Hammond and Axelrod’s paper^6^ except for the *Mobility* stage, which is new. For additional details, see the Methods section.
