# Supplementary material for: The Inevitability of Ethnocentrism Revisited: Ethnocentrism Diminishes As Mobility Increases
Source: Sci Rep. 2015 Dec 8;5:17963. doi: 10.1038/srep17963 (PMC4672305; doi:10.1038/srep17963)
Supplement: Supplementary Information [file srep17963-s1.pdf]

SUPPLEMENTARY INFORMATION

The Inevitability of Ethnocentrism Revisited: Ethnocentrism Diminishes As Mobility Increases

Soham De[1], Michele J. Gelfand[2], Dana Nau[3], Patrick Roos[4]

[1]Department of Computer Science, University of Maryland, College Park, MD, U.S.A.
Email: sohamde@cs.umd.edu

[2]Department of Psychology, University of Maryland, College Park, MD, USA.
Email: mgelfand@umd.edu

[3]Department of Computer Science and Institute for Systems Research, University of Maryland, College Park, MD, U.S.A.
Email: nau@cs.umd.edu

[4]Department of Computer Science, University of Maryland, College Park, MD, U.S.A.
Email: roos@cs.umd.edu

Evolutionary Dynamics of Hammond and Axelrod's Model

We confirmed the results of Hammond and Axelrod's model[1] by running simulations with their model. Supplementary Figure 1 shows a representative evolutionary trajectory obtained using their model, showing how the strategy proportions evolved. The four strategies are described near the beginning of the main paper: Cooperative (C), Defective (D), Ethnocentric (E) and Traitorous (T).

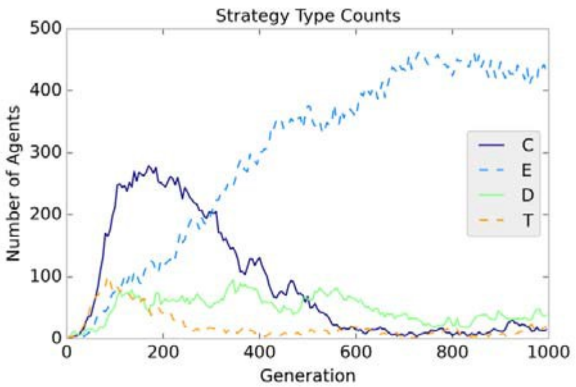

Supplementary Figure 1: A single simulation run showing the proportion of strategies in Hammond and Axelrod's model[1]. Ethnocentric (E) agents dominate after about 300 iterations, and population proportions stabilize after about 750 iterations.

Strategy Set

When an individual-entitative agent *i* interacts with an agent *j* that *i* has never encountered before, or when a group-entitative agent *i* interacts with an agent from a group that *i* has never encountered before, *i* must choose whether to cooperate or defect. That choice is part of *i*'s strategy; and in our experiments we allowed both possibilities. This doubled the total number of strategies in our simulations but made no meaningful difference in the results—so in favor of simplicity and clarity, we did not discuss this detail in the main paper.

Mutation Rate

In Hammond and Axelrod's model[1], during reproduction, an offspring will have the same strategy *s* as its parent, except that for each trait in *s*, there is a small probability μ=0.05 that this trait will be changed to a randomly chosen one. Notice that μ is not the probability that the offspring's strategy will differ from *s*. Instead, for each trait in *s*, it is the probability that this trait will be changed; and this happens independently for each of the traits in *s*. Consequently, the probability that an offspring retains the exact same strategy as its parent is inversely proportional to the number of possible traits.

Our model has a higher number of different possible traits than Hammond and Axelrod's model. Thus, in order to maintain roughly the same probability that an offspring will retain the same strategy as its parent, we needed to use a smaller value for μ.

In Hammond and Axelrod[1], each agent has 3 traits: the group tag and the actions to take when playing against an in-group and an out-group agent. However, in our model, the number of traits is significantly higher. Each group-entitative agent has 7 traits: the group tag, and traits specifying what action to take in each of the following six situations:

1. when an in-group agent cooperated on the last meeting,
2. when an in-group agent defected on the last meeting,
3. the first time one meets an in-group agent,
4. when an out-group agent cooperated on the last meeting,
5. when an out-group agent defected on the last meeting,
6. the first time one meets an out-group agent.

Similarly, each individual-entitative agent in our model has 4 traits. Thus, in order to ensure that the probability of an offspring retaining the same strategy as its parent is similar to that in Hammond an Axelrod's model, we needed to use a lower value for μ than what they used. We used μ=0.005.

Range Of Mobility

In our experiments, the reason we limited the mobility probability to $0 \leq m \leq 0.8$ is that cooperation breaks down at higher levels of mobility, as shown in Supplementary Figure 2(a). For example, at $m = 0.2$, about 80% of all actions are defections. Supplementary Figure 2(b) shows the reason for this breakdown. As m increases, the average number of games that an agent plays with the same opponent decreases monotonically. For example, at $m = 0.2$, each agent plays only 2 games with each opponent on average—which favors *AllD* rather than Tit-for-Tat (*TFT*).

As a societal analogy, agents are more likely to defect against each other if no agent interacts with any other agent long enough to create any kind of interpersonal ties. For example, consider the limiting case where mobility probability m=1.0, i.e., every agent moves to a different location in the grid at every iteration. This condition is similar to a *well-mixed population*, in which every agent can interact with every other agent in the population, with no network structure defining the set of possible interactions. Under well-mixed populations, it is well established that in a cooperation game like the Prisoner's Dilemma, the society devolves into universal Defection.

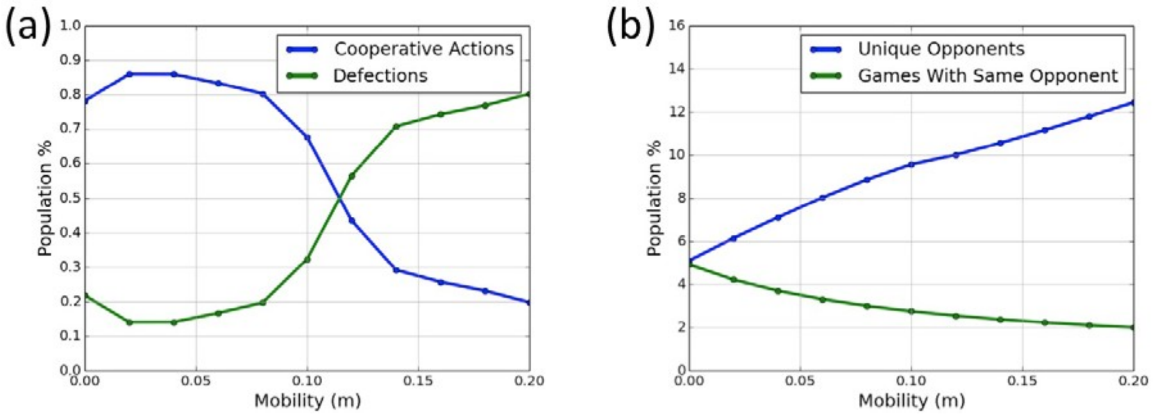

Supplementary Figure 2: Cooperation breaking down at higher mobility values. Each data point is an average of 100 individual simulation runs. The plots show (a) the proportion of agents cooperating and defecting; and (b) over an agent's lifetime, the average number of unique opponents it encounters, and the average number of games played against each of them.

Robustness Checks

To check robustness of the results of our model, we performed a series of additional experiments where we initialized the grid network with populations consisting of identical agents. We tried this with three different initial populations:

1. Each node on the grid is initialized with group-entitative agents of the same group tag, playing Cooperate with in-group agents, and Defect with out-group agents.
2. Each node on the grid is initialized with group-entitative agents of the same group tag, playing Cooperate with in-group agents, and TFT with out-group agents.
3. Each node on the grid is initialized with individual-entitative agents playing TFT against other agents.

We present the results of each different setting in Supplementary Figures 3, 4 and 5, respectively. For each case, we show a single simulation run showing the proportions of group-entitative vs. individual-entitative agents for mobility probability 0.0 and 0.05. We see that in each case, we exactly replicate the results of our model in the main paper, i.e., group-entitative agents dominate under conditions of no mobility, whereas individual-entitative agents evolve to dominate under conditions of higher mobility.

The reason for this is the exploration dynamics (or mutation phase) in our evolutionary game-theoretic model. In our model, an offspring can update each trait of its strategy to a randomly chosen one with probability μ=0.005. It is well established that in human cultures, individuals sometimes just test a new random behavior to see if it gives them higher payoff than their current behavior[3]. This *exploration dynamic* has been observed to be much more prevalent in cultural evolution than mutation is in biological evolution[2], and it is an integral part of evolutionary game theoretic models of cultural evolution. Because of the exploration dynamic, even when the grid starts with all agents playing a

particular strategy with clustering coefficient 1, a few agents will switch to new randomly chosen strategies. If some of these agents obtain higher payoffs than the rest of the population, other members of the population are more likely to change to their strategies. Because of this, our results (see Supplementary Figures 3, 4, and 5) show the population ultimately switching to the more profitable strategy regardless of the initial clustering of the population.

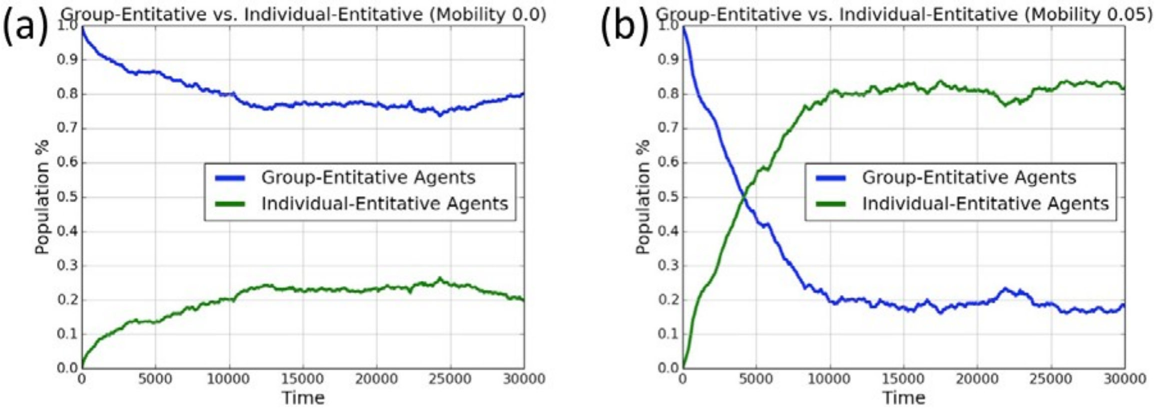

Supplementary Figure 3: Single simulation run for 30000 generations with mobility probability (a) *m*=0.0 and (b) *m*=0.05. Initial clustering of the grid: each node on the grid is initialized with group-entitative agents of the same tag, playing Cooperate with in-group agents, and Defect with out-group agents.

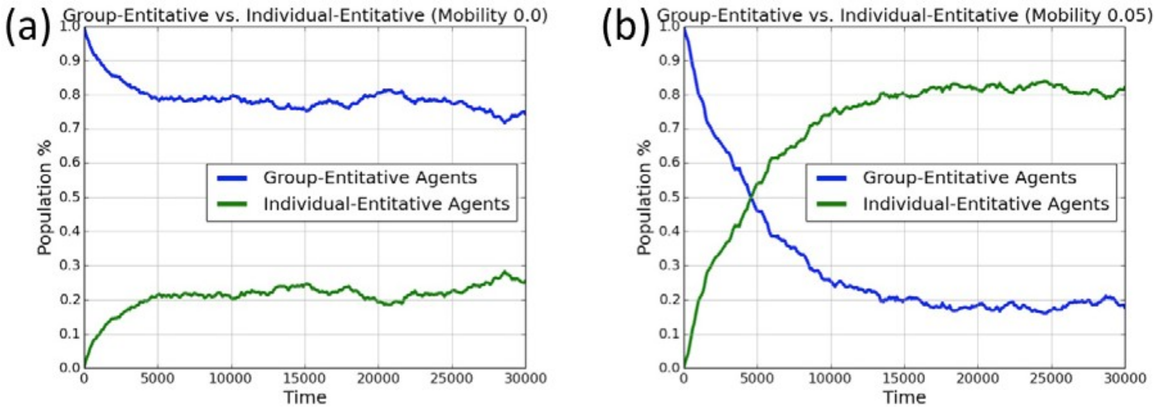

Supplementary Figure 4: Single simulation run for 30000 generations with mobility probability (a) *m*=0.0 and (b) *m*=0.05. Initial clustering of the grid: each node on the grid is initialized with group-entitative agents of the same tag, playing Cooperate with in-group agents, and TFT with out-group agents.

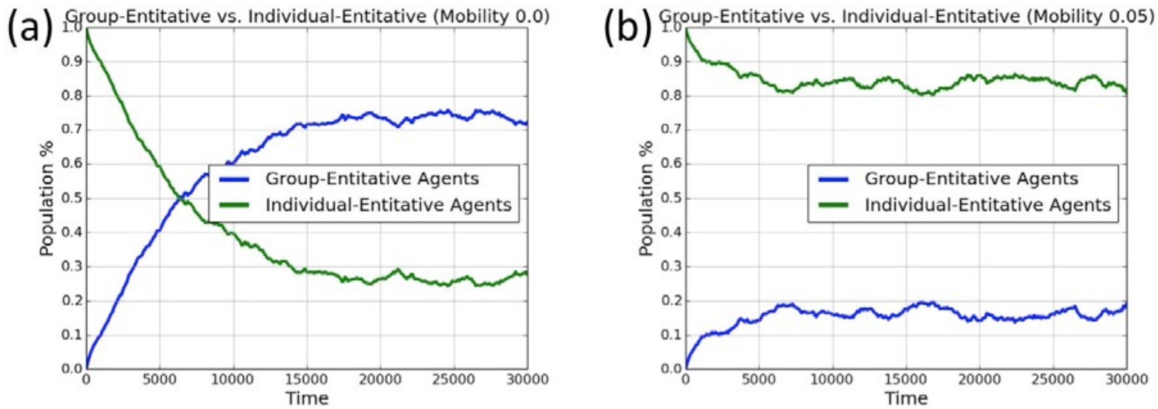

Supplementary Figure 5: Single simulation run for 30000 generations with mobility probability (a) *m*=0.0 and (b) *m*=0.05. Initial clustering of the grid: e ach node on the grid is initialized with individual-entitative agents playing TFT against other agents.

Code & Data

To enable reproducibility of the results presented in this paper, we have made available online the source code for running the simulations, the simulation data, and the empirical data presented in the paper. All three of these can be downloaded at the following URL: *https://umd.app.box.com/evolution-of-ethnocentrism-rev*

References

1. Hammond, R. A., & Axelrod, R. The evolution of ethnocentrism. *J. Confl. Resolut.* **50,** 926-936 (2006).
2. Traulsen, A., Hauert, C., De Silva, H., Nowak, M. A., & Sigmund, K. Exploration dynamics in evolutionary games. *Proc. Natl. Acad. Sci. U.S.A.* **106,** 709-712 (2009).
3. Traulsen, A., Semmann, D., Sommerfeld, R. D., Krambeck, H. J., & Milinski, M. Human strategy updating in evolutionary games. *Proc. Natl. Acad. Sci. U.S.A.* **107,** 2962-2966 (2010).
